# Ovarian Tumor Attachment, Invasion, and Vascularization Reflect Unique Microenvironments in the Peritoneum: Insights from Xenograft and Mathematical Models

**DOI:** 10.3389/fonc.2013.00097

**Published:** 2013-05-17

**Authors:** Mara P. Steinkamp, Kimberly Kanigel Winner, Suzy Davies, Carolyn Muller, Yong Zhang, Robert M. Hoffman, Abbas Shirinifard, Melanie Moses, Yi Jiang, Bridget S. Wilson

**Affiliations:** ^1^Department of Pathology, University of New MexicoAlbuquerque, NM, USA; ^2^Department of Biology, University of New MexicoAlbuquerque, NM, USA; ^3^Department of OB/GYN, University of New MexicoAlbuquerque, NM, USA; ^4^AntiCancer Inc., and Department of Surgery, University of California San DiegoSan Diego, CA, USA; ^5^Department of Physics, Institute of Biocomplexity, University of IndianaBloomington, IN, USA; ^6^Department of Computer Science, University of New MexicoAlbuquerque, NM, USA; ^7^Department of Mathematics and Statistics, Georgia State UniversityAtlanta, GA, USA

**Keywords:** ovarian cancer, tumor modeling, tumor microenvironment, metastasis, cellular Potts model, cell adhesion, angiogenesis, chemotaxis

## Abstract

Ovarian cancer relapse is often characterized by metastatic spread throughout the peritoneal cavity with tumors attached to multiple organs. In this study, interaction of ovarian cancer cells with the peritoneal tumor microenvironment was evaluated in a xenograft model based on intraperitoneal injection of fluorescent SKOV3.ip1 ovarian cancer cells. Intra-vital microscopy of mixed GFP-red fluorescent protein (RFP) cell populations injected into the peritoneum demonstrated that cancer cells aggregate and attach as mixed spheroids, emphasizing the importance of homotypic adhesion in tumor formation. Electron microscopy provided high resolution structural information about local attachment sites. Experimental measurements from the mouse model were used to build a three-dimensional cellular Potts ovarian tumor model (OvTM) that examines ovarian cancer cell attachment, chemotaxis, growth, and vascularization. OvTM simulations provide insight into the relative influence of cancer cell–cell adhesion, oxygen availability, and local architecture on tumor growth and morphology. Notably, tumors on the mesentery, omentum, or spleen readily invade the “open” architecture, while tumors attached to the gut encounter barriers that restrict invasion and instead rapidly expand into the peritoneal space. Simulations suggest that rapid neovascularization of SKOV3.ip1 tumors is triggered by constitutive release of angiogenic factors in the absence of hypoxia. This research highlights the importance of cellular adhesion and tumor microenvironment in the seeding of secondary ovarian tumors on diverse organs within the peritoneal cavity. Results of the OvTM simulations indicate that invasion is strongly influenced by features underlying the mesothelial lining at different sites, but is also affected by local production of chemotactic factors. The integrated *in vivo* mouse model and computer simulations provide a unique platform for evaluating targeted therapies for ovarian cancer relapse.

## Introduction

Ovarian cancer is often detected at a late stage of disease after the cancer has locally disseminated to the peritoneum. Visible tumors are surgically removed and residual microscopic disease is targeted with chemotherapy. However, 90% of patients who originally respond to treatment will relapse with chemotherapy-resistant disease (McGuire et al., 1996). Relapse is thought to occur because residual cancer cells aggregate in the peritoneal fluid and form microscopic tumor spheroids that are more resistant to chemotherapy (Shield et al., 2009). These spheroids can then adhere to the surface of organs in the peritoneum and seed new tumors, encouraged by chemokines and growth factors within the peritoneal fluid (Milliken et al., 2002; Bast et al., 2009).

A common feature of the peritoneal environment is the mesothelial lining that cancer cells must sequentially bind to (Strobel and Cannistra, 1999; Casey et al., 2001; Kenny et al., 2008) and penetrate (Burleson et al., 2006; Iwanicki et al., 2011) in order to adhere to underlying tissues. Recent *in vitro* studies suggest that this penetration step can occur within a few hours after spheroid attachment (Iwanicki et al., 2011). Nevertheless, unique features associated with different organs clearly influence progression in this disease. For example, ovarian cancer cells preferentially colonize the omentum, a fatty tissue that has pockets of resident immune cells referred to as “milky spots” and easily accessible blood vessels (Gerber et al., 2006; Khan et al., 2010; Nieman et al., 2011). Cancer cells also colonize other organs in the peritoneum, with distinct growth rates and morphology depending on the site. It is reasonable to expect that these heterogeneous tumor populations will respond differently to treatment, motivating further investigation into the features of the microenvironment that govern these differences.

To establish a mouse model of ovarian cancer relapse, SKOV3.ip1 cells expressing fluorescent proteins [GFP, red fluorescent protein (RFP)] were injected into the peritoneum of nude mice and the resulting tumors growing on the omentum, intestine, mesentery, and spleen were imaged. Excised tumors were processed for both transmission and light microscopy, providing detailed information about the cellular environment and vascularization patterns.

The distinct features in tumor morphology at different sites led us to consider the potential contributions of local chemotactic factors, oxygenation and adhesion through mathematical modeling. In recent years, mathematical models have moved beyond the generic models of tumor growth and development (e.g., Jiang et al., 2005; Shirinifard et al., 2009; Morton et al., 2011; Giverso and Preziosi, 2012) and are now able to realistically model cancers, e.g., breast cancer (Chauviere et al., 2010; Macklin et al., 2012) and colon cancer (Dunn et al., 2012). Few have addressed the unique features of ovarian cancer. Arakelyan et al. (2005) modeled ovarian tumor growth response to the dynamics of vascular density and vessel size (Arakelyan et al., 2005). Giverso et al. (2010) developed a two-dimensional model of early ovarian tumor spheroid invasion through the mesothelium and underlying extracellular matrix (ECM) (Giverso et al., 2010).

In the present work, our focus is on understanding the distinct features of tumor morphology at different sites in ovarian cancer relapse in three dimensions. The cellular Potts model framework was chosen because of its previous successes in studying similar problems in tumor growth and angiogenesis (Jiang et al., 2005; Shirinifard et al., 2009). Our cell-based and geometrically realistic ovarian tumor model (OvTM), takes into account characteristics of the peritoneal microenvironment and provides insight into the earliest steps in spheroid attachment, invasion, and vascularization within the peritoneum. In particular, homotypic and heterotypic adhesion observed between SKOV3.ip1 xenograft cancer cells and the niche tissue structure are the starting point of OvTM. We applied the model to explore the roles of cell adhesion, cell migration and proliferation as influenced by the microenvironment at two sites and were able to reproduce experimental observations. The ultimate goal of our model is a realistic representation of spheroid growth, whose dimensions and morphology qualitatively resemble the tumors disseminated in different tissue niches in the peritoneal cavity in our mouse xenografts. Such a model can be further developed to include short-term drug delivery after debulking surgery, allowing the evaluation of local drug responsiveness.

## Materials and Methods

### Cell culture and cell lines

SKOV3.ip.1 parental cells and GFP-stable transfectants were kind gifts of Laurie Hudson and Angela Wandinger-Ness (UNM). This aggressive line was passaged through a nude mouse (Yu et al., 1993). Cells were maintained in RPMI-1640 medium supplemented with 5% heat-inactivated FBS, 1% l-glutamine, 1% sodium pyruvate, and 0.5% penicillin/streptomycin (Invitrogen, Grand Island, NY, USA). SKOV3.ip1-GFP cells were treated with 250 μg/ml hygromycin to maintain GFP expression. To create RFP-expressing SKOV3.ip1 cells, parental cells were transfected with the pTagRFP-N vector (Axxora, San Diego, CA, USA) using Lipofectamine LTX reagent (Invitrogen). Stably fluorescent cells were selected with geneticin sulfate (Invitrogen) for 1 week. Transfectants were sorted for high fluorescence using a Beckman Coulter Legacy MoFlo cell sorter (UNM Flow Cytometry Core Facility).

### Intraperitoneal mouse model of ovarian cancer relapse

All mouse procedures were approved by the University of New Mexico Animal Care and Use Committee, in accordance with NIH guidelines for the Care and Use of Experimental Animals.

Nu/nu nude mice (NCI) or nude mice ubiquitously expressing RFP (Anticancer Inc., San Diego, CA, USA) (Yang et al., 2009) were engrafted by intraperitoneal injection with 100 μl of a single cell suspension containing five million SKOV3.ip1 cells expressing GFP. Tumor adhesion and invasion was assessed at 4 days and 2 weeks post-injection. For low magnification assessment of total tumor burden, eight nude mice were imaged using a Pan-A-See-Ya Panoramic Imaging System (Lightools, Inc., Encinitas, CA, USA) 2 or 3 weeks post-injection of SKOV3.ip GFP cells. For improved resolution images (up to 16×, single cell resolution), mice were imaged on the OV100 Olympus whole mouse imaging system (Olympus Corp., Tokyo, Japan) at AntiCancer, Inc., San Diego, CA, USA, as previously reported (Yamauchi et al., 2006).

Where described, sections of intestine and attached mesentery with tumors were excised (Fu and Hoffman, 1993) and fixed in zinc fixative for 30 min (Howdieshell et al., 2011). Samples were mounted on glass slides with ProLong Gold mounting media (Invitrogen). GFP fluorescence and brightfield images were collected on a Nikon TE2000 Microscope (UNM Microscopy Core Facility) using an Axiocam digital color camera and SlideBook Image Acquisition software.

### Co-injection experiments

SKOV3.ip1-GFP cells and SKOV3.ip1-RFP cells (2.5 × 10^6^ each population) were harvested from culture by trypsinization and mixed together as a single cell suspension immediately before injecting a total of five million cells into the peritoneum of three nude mice. For consecutive injections, 2.5 million SKOV3.ip1-GFP cells were injected IP into three nude mice followed by injection of 2.5 million SKOV3.ip1-RFP cells a week later. The mice were sacrificed at the end of week 2 and tumors were imaged on the OV100.

### Histology and immunofluorescence

Mouse tumors were fixed in formalin or zinc fixative, embedded in paraffin, sectioned and hematoxylin/eosin (H&E) stained by TriCore (Albuquerque, NM, USA) or processed for immunofluorescence using anti-CD31 antibodies (BD Biosciences, San Jose, CA, USA). Images were acquired on a Zeiss AxioSkop or LSM500 confocal microscopes. The area of mesenteric tumors was determined by analysis of images from H&E-stained sections using ImageJ (Schneider et al., 2012). The cross-sectional tumor area corresponding to the hypoxic threshold was calculated to be <104,000 μm^2^ based on the diameter of the spheroid in Figure 7B (364 μm).

### Transmission electron microscopy

Tissue was collected and fixed in 2% glutaraldehyde, post-fixed with osmium tetroxide, dehydrated in ascending alcohols, and embedded in Epon. Ultra-thin sections were stained and imaged on a Hitachi H600 transmission electron microscope (TEM). To identify SKOV3.ip1 cells present in tissue samples, their characteristic nuclear ultrastructure was determined from high magnification TEM images taken of SKOV3.ip1-GFP cells grown as 5,000 cell spheroids in a *U*-bottom Lipidure-coated 96-well plate (NOF America, Irvine, CA, USA) for 48 h.

### The ovarian tumor model

We based our model on the following set of major assumptions that are inspired by the biology and empirical data.

1. The three-dimensional (3-D) tissues we model consist of ovarian tumor (SKOV3.ip1), mesothelial, adipocyte, endothelial, and smooth muscle cells, as well as ECM fibers and peritoneal fluid.
2. Adhesion strengths between various cell types are prescribed and remain constant, e.g., adhesion between SKOV3.ip1 cells is stronger than between SKOV3.ip1 and mesothelial cells.
3. The chemical environment consists of oxygen, tumor-secreted VEGF, adipocyte-secreted IL-8 (Nieman et al., 2011), and another unidentified growth factor (Growth Factor 2) secreted by blood vessels.
4. Both IL-8 and Growth Factor 2 are chemoattractants for tumor cells. VEGF is a chemoattractant for endothelial cells. Chemotaxis speed is proportional to the chemical gradient.
5. Cells consume oxygen supplied by the peritoneal fluid and blood vessels. When oxygen levels are below a threshold value (20 mm Hg), tumor cells become hypoxic and stop growing. They resume proliferation if oxygen rises above the threshold level.
6. Cells are required to approximately double in volume before dividing and their division times have a Gaussian distribution.
7. Ovarian tumor model does not represent the flow of peritoneal fluid (or ascites). Because the diffusion process for all chemicals considered in our study is much faster than the cellular processes under consideration, chemicals are well-mixed. Therefore, it is reasonable to omit convective delivery of chemicals in this model.
8. Cells can become immobilized if we impose shape constraints. Addition of a surface area constraint as well as a high volume constraint can make cells insensitive to changes in adhesion and to low chemotaxis constants. Adhesion parameter sensitivity analysis was therefore conducted without a surface area constraint on the cells. This constraint was added after adhesion optimization to produce cells that were more spheroidal, as they are seen *in vivo*.

The 3-D ovarian tumor growth and invasion model, OvTM, is based on the cellular Potts model framework using CompuCell3D (Cickovski et al., 2007; Swat et al., 2009). Ovarian cancer cells growing in the peritoneum were simulated using parameters obtained from the mouse model and from published data. Parameters for the SKOV3.ip1 cells, endothelial cells, oxygen, growth factors, and measurements of mouse peritoneal tissue are shown in Table 1.

**Table 1:** **OvTM parameters**.

	Value	Units	Source
**CHEMICAL FIELDS**			
O_2_ concentration (blood and peritoneal fluid)	98.5	mm Hg	Shirasawa et al. (2003), Kizaka-Kondoh et al. (2009)
O_2_ diffusion (*D*_O_2__)	84,000	μm^2^/min	MacDougall and McCabe (1967)
VEGF diffusion (*D*_V_)	600	μm^2^/min	Serini et al. (2003), Bauer et al. (2009)
VEGF decay (γ*_V_*)	0.01083	/min	Serini et al. (2003), Bauer et al. (2009)
VEGF secretion: normoxic tumor cell (α*_V_*)	3.82 × 10^−7^	pg/min/cell	Huang et al. (2000)
Chemotactic Factor 2 diffusion (*D*_C_2__)	700	μm^2^/min	e Serini et al. (2003), Bauer et al. (2009)
Chemotactic Factor 2 decay (κ_C_2__)	0.01083	/min	e Serini et al. (2003), Bauer et al. (2009)
Chemotactic Factor 2 secretion (α_C_2__)	1.8 × 10^−4^	pg/min/cell	e Serini et al. (2003), Bauer et al. (2009)
IL-8 diffusion (*D*_C_1__)	15000	μm^2^/min	Li Jeon et al. (2002)
IL-8 decay (equal to VEGF) (ρ_C_1__)	0.01083	/min	e as in Jain et al. (2008)
IL-8 secretion by visceral adipocyte (α_C_1__)	2.2 × 10^−4^	pg/min/cell	Bruun et al. (2004)
IL-8 background concentration (peritoneal fluid)	1.732	pg/ml	Barcz et al. (2002)
**METABOLIC PARAMETERS**			
O_2_ consumption: proliferating cancer cell (ε)	4.93	fmoles/min/cell	Freyer and Sutherland (1985), Casciari et al. (1992)
O_2_ threshold for hypoxia and VEGF production	19	mm Hg	Höckel and Vaupel (2001), Evans et al. (2006), Shirinifard et al. (2009)
VEGF activation threshold for angiogenesis	0.0001	pg/cell volume	e
VEGF deactivation threshold for angiogenesis	0.00002	pg/cell volume	e
**RATE PARAMETERS**			
SKOV3.ip1 invasion speed	10	μm/h	e from Iwanicki et al. (2011)
Rate of ECM degradation	0.55	μm^2^/min	Bauer et al. (2009)
SKOV3.ip1 cell cycle duration	25.5 ± 1	h	m
Vascular endothelial cell cycle duration	24 ± 1	h	Ausprunk and Folkman (1977), Levine et al. (2001), Bagley et al. (2003)
Cell volume after division	96 ± 17	%	e from m
**MORPHOMETRIC PARAMETERS**			
Cancer cell radius	3.50	μm	m
Average adipocyte cell radius	10.1	μm	m
ECM (extracellular matrix) thickness	2	μm	m
ECM collagen fiber radius	1	μm	m
ECM collagen fiber length	20	μm	e
Vascular endothelial cell diameter (initial size)	10	μm	Bauer et al. (2009)
Height of mesothelial cell on mesentery	0.44–2.5	μm	m, Khanna and Krediet (2009)
Average distance between adipocytes in the mesentery	0.2	μm	m

*Values were obtained from direct measurement (indicated by “m”) of tissues and tumors or from previously published work. Where experimental values were not available, values were estimated for the model (indicated by “e”)*.

In the OvTM, five cell types are considered: ovarian tumor (SKOV3.ip1), mesothelial, adipocyte, endothelial, and smooth muscle. ECM fibers and peritoneal fluid are represented as special types. This cell-based model describes cell growth, division, death, and chemotactic migration within a 3-D tissue environment that mimics the specific organ site.

Cells are domains on a 3-D lattice. Each cell has an ID number, *S*, on each lattice site *i* of the cell domain, and an associated cell type τ. Cell–cell and cell–environment interactions are specified by an “*effective energy*”:

(1) $$\begin{array}{rcll} H & =\underset{i,j}{\sum }J_{\tau }\left(S_{i}\right),\tau \left(S_{j}\right)\left[1-\delta \left(S_{i},\;S_{j}\right)\right] & & \\ & \;+\underset{S}{\sum }{\lambda \left[V\left(S\right)-V^{t}\left(S\right)\right]}^{2}+\underset{i}{\sum }\mu C_{i}. & \end{array}$$

The parameter *J* describes the cell-type-dependent adhesion. Adhesion coefficients (*J*) for the cell types in each model are listed in Tables 2 and 3. The Kronecker delta function δ ensures no energy is within cells where ID numbers are the same, limiting adhesion to cell boundaries. A cell’s volume *V* is elastically constrained to *V^t^*, the target cell volume; *V^t^* is constant for the tissue cells, but is set to increase linearly for proliferating cells. μ is the chemical potential describing the strength of chemotaxis, and *C* is the chemical concentration at the cell. This term applies to cancer and endothelial cells when their respective chemoattractant signals are above threshold activation values.

**Table 2:** **Combined tension and adhesion matrix for OvTM simulations of spheroid invasion, growth and angiogenesis**.

	PF	VM	PTC	SE	VW	A	ECM	SM
PF	0	10	10	10	10	10	10	10
VM	10	0	20	20	10	20	0	10
PTC	10	20	0	0	0	3	3	0
SE	9.5	19.5	−0.5	1	0	13	5.5	2.5
VW	10	10	0	0	0	13	0	0
A	3.5	13.5	−3.5	6.5	6.5	13	1	0
ECM	9.5	−0.5	2.5	0.5	−0.5	0.5	1	0
SM	10	10	0	2.5	0	0	0	0

*White boxes show adhesion coefficients between cell types (*J*_ij_) and green boxes show adhesion coefficients between cells of the same type (*J*_ii_). Gray boxes show the surface tension values between cell types. Surface tension is defined as ST_ij_ = *J*_ij_−(*J*_ii_ + *J*_jj_)/2 (Glazier and Graner, 1993). Negative ST_ij_ signifies attraction, and positive ST_ij_ signifies repulsion. Surface tension = 0 for all ST_ii_, but homotypic adhesion *J*_ii_ can still be strong. Maximum adhesion is 0. Mesenteric invasion models contain PF, peritoneal fluid; VM, visceral mesothelium; PTC, proliferating tumor cells; VW, vessel wall endothelial cells, A, adipocytes; ECM, extracellular matrix (ECM). Included in the spheroid growth model are: PTC, proliferating cancer cell; PF, peritoneal fluid, VM, visceral mesothelium, VW, vessel wall endothelial cells; ECM, extracellular matrix; SM, smooth muscle. Models with angiogenesis further include VW, vessel wall endothelial cells; SE, sprouting endothelial cells*.

**Table 3:** **Exploration of adhesion parameters in spheroids attached to the surface of the small intestine**.

Parameters tested			Results	Spheroid images	
				2-D (tumor center)	3-D
Low homotypic adhesion and low heterotypic adhesion to non-tumor cells			By 24 h, tumor cells show reduced volume and the spheroid is no longer cohesive	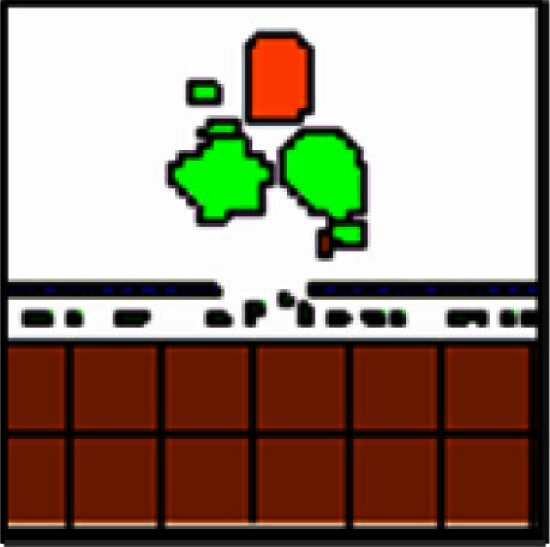	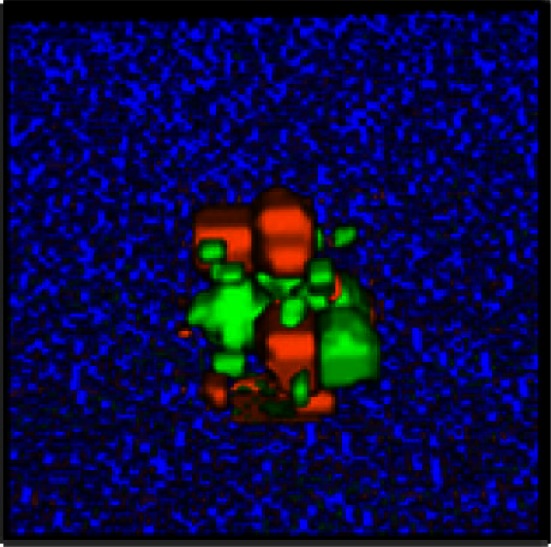
Cell type 1	Cell type 2	Adhesion 1 ⇔ 2			
PTC	PTC	20			
PTC	other	20			
other = PF, ECM, VM, and SM					
Low homotypic adhesion and high heterotypic adhesion			By 24 h, the spheroid has fragmented while the mesothelium has aggregated	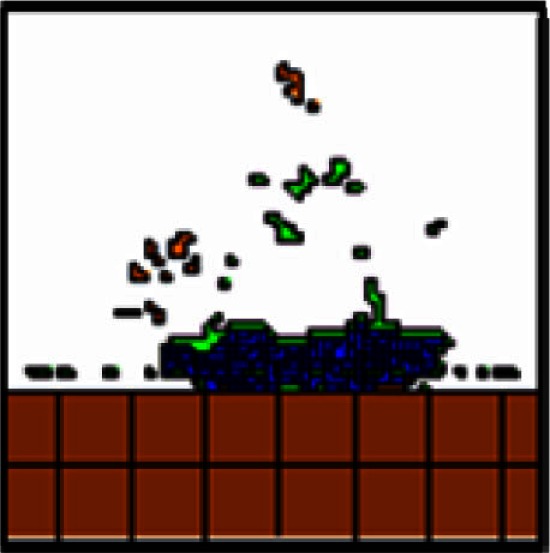	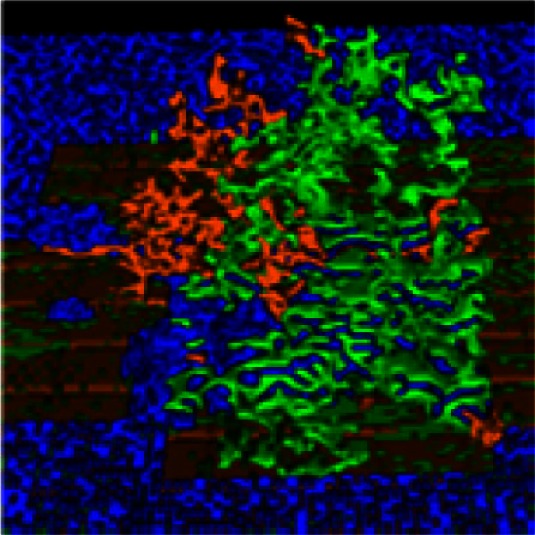
Cell type 1	Cell type 2	Adhesion 1 ⇔ 2			
PTC	PTC	20			
PTC	other	Tested 0, 1, 3, and 5			
High homotypic adhesion and high heterotypic adhesion			By 12 h, the spheroid has begun to disintegrate and the mesothelium has aggregated at the base of the spheroid	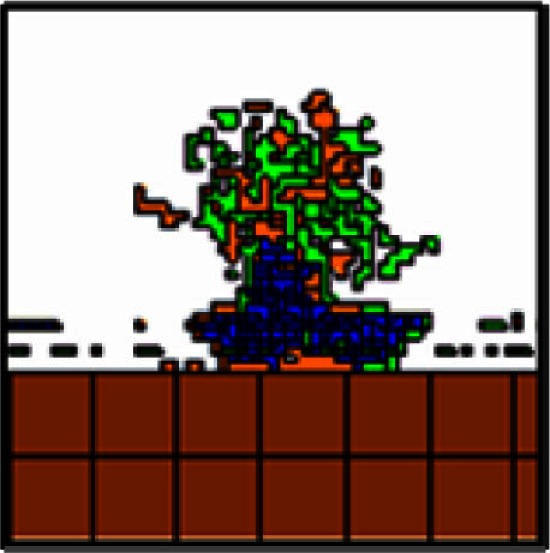	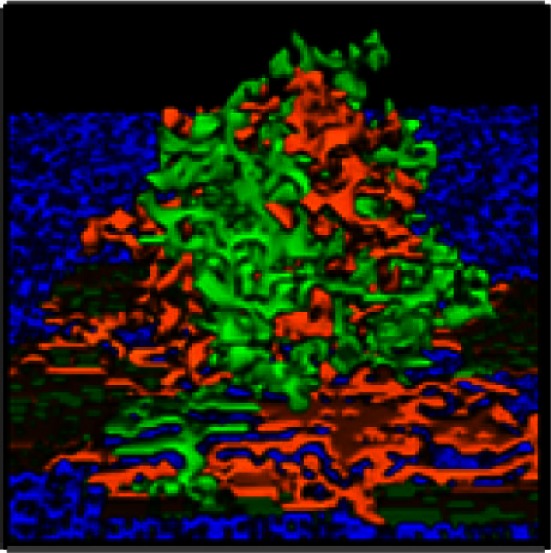
Cell type 1	Cell type 2	Adhesion 1 ⇔ 2			
PTC	PTC	0			
PTC	other	0			
High homotypic adhesion and low heterotypic adhesion No surface area constraint			At 24 h, a coherent spheroid with a similar appearance to the SKOV3.ip1 *in vivo* tumors can be seen. The mesothelium remains intact. However, cells are abnormally convoluted	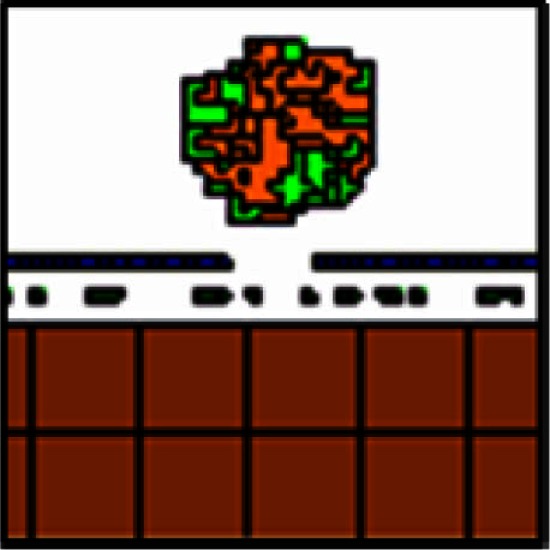	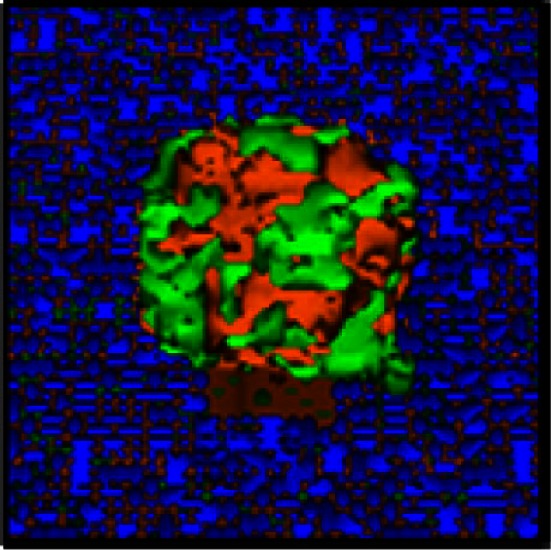
Cell type 1	Cell type 2	Adhesion 1 ⇔ 2			
PTC	PTC	0			
PTC	other	Tested 10 and 20			
High homotypic adhesion and low heterotypic adhesion Additional surface area constraint			This model most closely approximates the shape of SKOV3.ip1 spheroids and cells *in vivo* as shown at 12 h	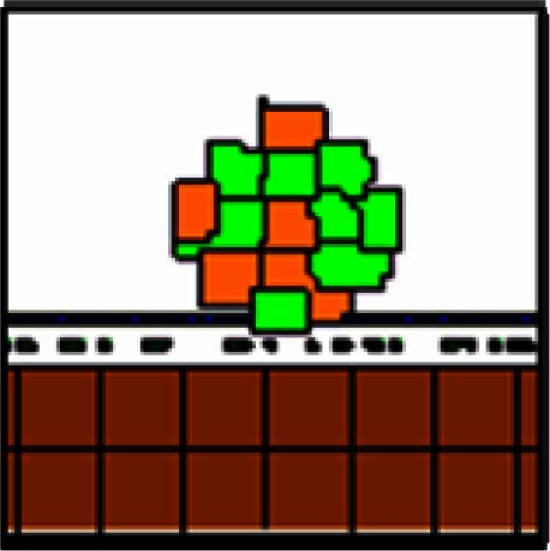	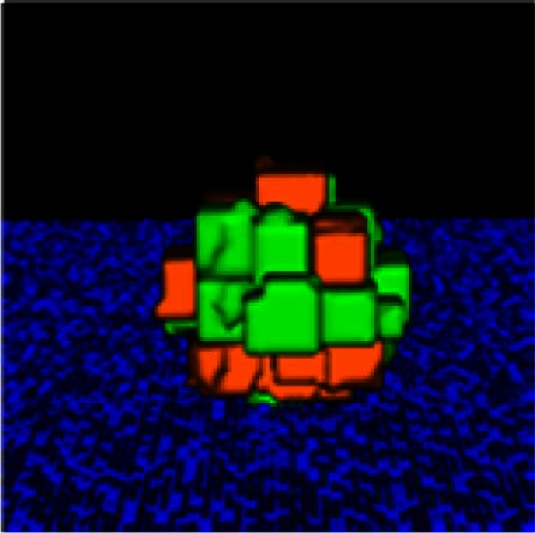
Cell type 1	Cell type 2	Adhesion 1 ⇔ 2			
PTC	PTC	0			
PTC	other	See Table 2			

*Abbreviations are the same as in Table 2*.

A modified Metropolis algorithm was used to simulate cell dynamics. A cell boundary lattice site is selected at random; the cell number, *S*, is copied to an unlike neighbor site *S*′ (selected at random). This copying corresponds to the cell *S* protruding a unit volume into the neighboring cell *S*′. The difference between the effective energies before and after the protrusion event, *E*, determines if this copying event will be accepted. If the energy decreases, the protrusion is accepted; if it increases, the protrusion is accepted with a Boltzmann probability, exp(−*E/T*). The effective temperature, *T*, describes the amplitude of cytoskeletal fluctuation (Mombach et al., 1995). By such microscopic membrane protrusion and retraction, the cells perform biased random walks, and rearrange themselves, within the constraints of their volumes and chemical guidance.

#### Chemical dynamics

Chemical dynamics are evaluated through continuous diffusion equations and are considered well-mixed in the peritoneal fluid. Chemicals acting as chemoattractants to SKOV3.ip are: adipocyte-secreted IL-8 (Nieman et al., 2011), and an unidentified growth factor secreted from the vessel (Growth Factor 2). The chemoattractant for endothelial cells during angiogenesis is VEGF. A system of partial differential equations describes the chemical dynamics, including diffusion through tissues, growth factor decay, glucose and oxygen consumption, and cell-uptake of signal molecules.

(2) $$\begin{array}{lll} \frac{\partial C_{1}}{\partial t} & =D_{C_{1}}\nabla^{2}C_{1}-\rho_{C_{1}}C_{1}+\alpha_{C_{1}}\delta \left[\tau \left(S\right),\text{adipocyte}\right], & \end{array}$$

(3) $$\begin{array}{lll} \frac{\partial C_{2}}{\partial t} & =D_{C_{2}}\nabla^{2}C_{2}-\kappa_{C_{2}}C_{2}+\alpha_{C_{2}}\delta \left[\tau \left(S\right),\text{vessel}\right], & \end{array}$$

(4) $$\begin{array}{lll} \frac{\partial V}{\partial t} & =D_{V}\nabla^{2}V-\gamma_{V}V+\alpha_{V}\delta \left[\tau \left(S\right),\text{tumor}\right], & \end{array}$$

where *C_1_* is IL-8, *C_2_* is Growth Factor 2, and *V* is VEGF. These equations describe diffusion through tissue, decay, and production by source cells.

Oxygen is delivered by the vessel (see Figure 7) and diffuses from the peritoneal fluid. Its level is kept constant within the peritoneum. Diffusion of O_2_ through tissue and its consumption by the cells is described as:

(5) $$\frac{\delta O_{2}}{\delta t}=D_{O_{2}}\nabla^{2}O_{2}-\in \delta \left[\tau \left(S\right),\text{tumor}\right]$$

Depending on the availability of oxygen in their surrounding environment, cancer cells have the potential to be proliferating, quiescent or necrotic. Although the capability for cancer cells to become hypoxic or necrotic was integrated into the model, the spheroids we simulated were too small in diameter to develop internal hypoxia under normal physiological conditions, and never became hypoxic or necrotic.

The secretion rates for IL-8, VEGF, and Chemotactic Factor 2 were taken from quantitative experiments (or estimated for Chemotactic Factor 2). Oxygen was kept constant at the simulation boundaries with the value corresponding to the steady-state oxygen level in the mouse peritoneal fluid (∼98.5 mmHg). Background IL-8 was also kept constant at the *z* boundaries, defined as normal to the mesothelium surface, with the value corresponding to its steady-state concentration in the peritoneal fluid. IL-8 concentration at the *x* and *y* boundaries was constant at levels diffusing from the adipocytes. Boundary values of VEGF and Chemotactic Factor 2 were set to steady-state at 0, as background values were unknown and the cells generated small, concentrated fields that reached approximately 0 at the distance of the simulation boundaries. For the diffusion rates of these four chemicals (*D* > 15 μm^2^/min), CompuCell3D cannot generate boundary conditions for tissues or cell types, as the necessary solver requires extra solutions per time step that make running models intractably slow (e.g., ∼525,000 extra solutions per time step are required in the case of oxygen, resulting in ∼24 h simulation time/1 min experimental time simulated). All parameters are listed in Table 1.

The chemotaxis constant for SKOV3.ip1 cell response to IL-8 was increased until the cells were moving through the IL-8 gradient in mesenteric fat tissue at a rate we derived from the ovarian cancer spheroid experiments by Iwanicki et al. (2011). Chemotaxis by tumor cells to Chemotactic Factor 2 coming from the vessel was tuned to have the same order of magnitude of response as to IL-8 (order of magnitude (IL-8 concentration × chemotaxis constant) = order of magnitude (Chemotactic Factor 2 concentration × chemotaxis constant), where the chemotaxis constant = 1 × 10^13^.

#### Cell reproduction

In OvTM, the cellular level model and the chemical environment model are integrated through the choice of a common time-scale. In the cellular Potts model, each Monte Carlo Step (MCS) corresponds to as many cellular protrusion events as the number of lattice sites in the simulation domain. We define one MCS to correspond to 1 min of real time. The chemical equations (Eqs 2, 3, and 4) are solved at the time step of 1 min, when the cell lattice configuration and states are assumed constant. The cells update their states according to their local chemical concentrations. The cell lattice is then updated for one MCS assuming chemicals stay constant. Such iterative feedback and update links the discrete cellular Potts model and the continuous chemical equations together. Each cell has its own division time and age clock. Division time for cells is set on a Gaussian distribution; SKOV3.ip1 cells divide between 24.5 and 26.5 h and endothelial cells divide between 23 and 25 h. A cell divides when two conditions are satisfied: (a) the cell’s age is greater than or equal to its division time, and (b) the cell’s volume is greater than or equal to its target volume, which increases linearly with the cell’s age. When the cell (*S*) divides, it is halved into two daughter cells, *S* and *S*′.

#### Tissue microenvironments

The three OvTM model scenarios each represent several cubic millimeters of peritoneal space, about one third of which is tissue. (The invasion model is 7.6 mm^3^ and contains 2.6 mm^3^ of tissue.) The rest of the volume is filled with peritoneal fluid present in the mammalian peritoneal cavity. Cell shapes and sizes were determined by cell morphometry studies of normal mice, nude mice, and SKOV3.ip1 xenografts. Thickness of the adipose layer surrounding vessels of the mesentery was taken from the literature. Tissue rigidity in smooth muscle and adipocytes was estimated based on cell junctions and spacing from EM images of mouse tissues. The depth of penetration of SKOV3.ip1 cells on both tissue types was based upon xenograft tumors in our microscopic images.

The tissue microenvironments we considered were the outer surface of the intestine (Figures 2 and 7) and the mesentery (Figure 5). A layer of mesothelium covers both surfaces. Both environments are initialized with a tumor spheroid of seven cells contacting the center of the contiguous mesothelial surface in the peritoneal cavity.

On the intestine, smooth muscle lies beneath the mesothelium, separated from it by a thin layer of ECM. Ovarian cancer cells can push aside the mesothelium and degrade ECM (Kenny et al., 2008; Sodek et al., 2008), but strong adhesion between the muscle cells prevents further invasion. Scattered blood vessels lie just below the mesothelium. In our model, initiation of angiogenesis was triggered by a threshold VEGF level. When the local VEGF concentration exceeds a threshold, endothelial cells lining the blood vessels begin to proliferate and migrate, organizing into vascular sprouts.

Tumors on the omentum or mesentery can invade through the mesothelium and migrate through the loose matrix and adipocyte fields below. On the mesentery, ovarian cancer cells adhering to the mesothelium push past the mesothelial cells as chemotactic gradients originating from adipocytes and vasculature stimulate migration into the tissue.

Both models are initialized with a tumor spheroid of seven cells contacting the center of the contiguous mesothelial surface in the peritoneal cavity. We assume ECM degradation rate by SKOV3.ip1 tip cells at the invading front is the same as the degradation rate by endothelial cells at the sprout tip (Bauer et al., 2009).

The process of angiogenesis is driven by endothelial cell chemotaxis toward VEGF, and by differential adhesive interactions between endothelial sprout cells and cancer cells. This simple model suggests that, to produce vasculature morphology similar to that in very small xenograft tumors, latent endothelial cells must become proliferating endothelial cells and initiate angiogenic behavior as soon as the spheroid comes within diffusion distance for low concentrations of VEGF of the vessel. Given VEGF production of 3.82 × 10^−7^ pg/min/SKOV3.ip1 cell, the threshold for the switch from latent to sprouting endothelial cells is set at 2.08 × 10^−8^ pg/cell volume, which initiates angiogenesis when the spheroid is about 5 μm from the vessel.

#### Exploration of adhesion parameters

To reproduce the morphology of *in vivo* SKOV3.ip1 xenografts on the intestine, we tested four combinations of homotypic and heterotypic adhesion parameters (Table 3). Only when tumors were parameterized to have high homotypic adhesion between cancer cells along with low heterotypic adhesion between cancer cells and components of the microenvironment (peritoneal fluid, ECM, visceral mesothelium, and smooth muscle cells) did cancer cells form rounded spheroids similar to those seen *in vivo*. A surface area constraint was also imposed on the cancer cells to maintain cell integrity. We ran at least five replica simulations for each parameter set, and observed no qualitative differences, within small variations, in our results.

## Results

### SKOV3.ip1 ovarian cancer cells adhere to the surface of numerous organs in the peritoneum and form large tumors by 2 weeks post-injection

**Figure 1:**
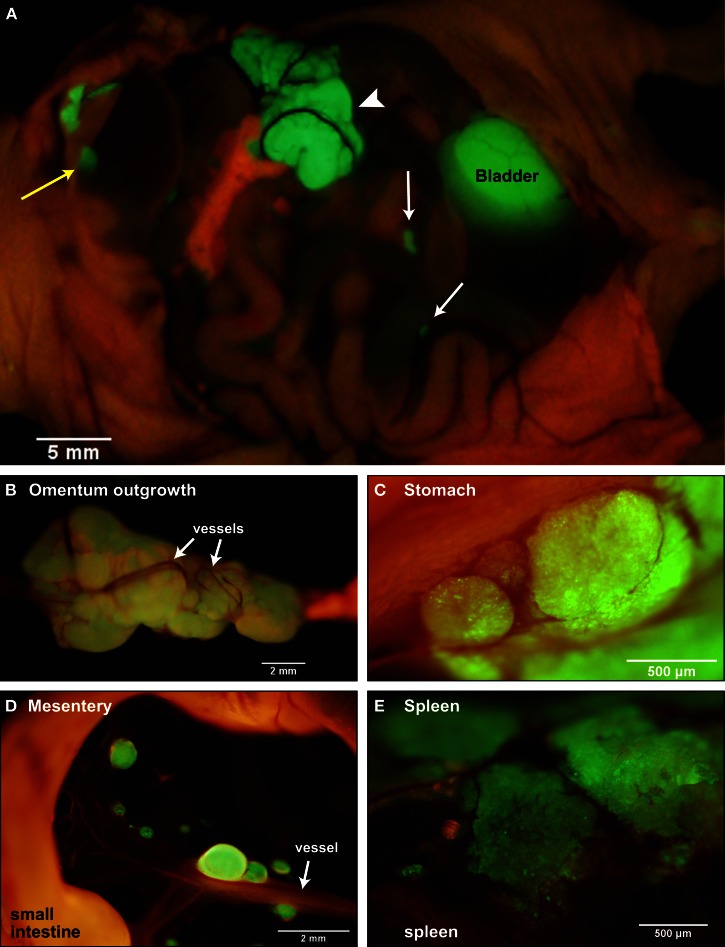
**SKOV3.ip1-GFP cells colonize the surface of many organs in the mouse peritoneum**. **(A)** Image of SKOV3.ip1 tumors growing from the omentum (large central tumor, arrowhead), the intestine, attached mesentery (white arrows), and the liver (yellow arrow) of an RFP nude mouse. Tumors on the spleen are not visible in this image. **(B)** The largest tumors are attached to the omentum located on the larger curvature of the stomach and are well vascularized. **(C)** Tumors attached to the stomach are spherical and non-invasive. **(D)** On the mesentery, small tumors are located adjacent to major blood vessels (arrow). **(E)** Small tumors growing on the spleen have a flatter, sponge-like morphology with less well defined borders between the tumor (green) and the normal tissue.

To recapitulate the essential steps in ovarian cancer relapse from minimal residual disease in the peritoneum, five million SKOV3.ip1 human ovarian cancer cells expressing GFP were injected into the peritoneum of nude mice as a single cell suspension. Mice were euthanized after 2 weeks and mounted on the stage of an OV100 fluorescence imaging system. As shown in Figure 1A, macroscopic tumors developed on numerous organs in the peritoneum during this period. The main sites of attachment were the omentum (Figures 1A,B), the surface of the stomach (Figure 1C) and small intestine, the mesentery (Figure 1D) and the spleen (Figure 1E). Tumors also developed on the liver in half of the mice examined. The largest tumors grew from the omentum and expanded into the peritoneum, reaching volumes up to 200 mm^3^ by 2 weeks. Even within this short time frame, the tumors are well vascularized with vessels penetrating and wrapping around the outside of the largest tumors. Many smaller tumors can be found attached to the mesentery, often visible to the naked eye, ranging in size from 0.4 μm^3^ to 1 mm^3^.

### Strong homotypic interactions drive SKOV3.ip aggregation

**Figure 2:**
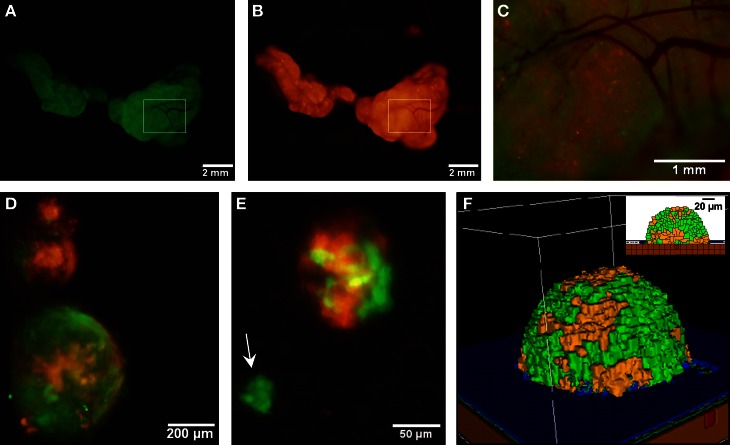
**Co-injected SKOV3.ip1-GFP and RFP cells yield chimeric tumors**. Equal numbers of SKOV3.ip1-GFP and SKOV3.ip1-RFP cells were injected as a single-cell suspension into the peritoneum of nude mice. **(A–C)** Large tumors on the omentum are both GFP-positive (GFP-filter) **(A)** and RFP-positive (RFP-filter) **(B)**. White boxes: magnified region shown in **(C)**. **(C)** A 5X magnified composite image of the tumor from **(A,B)** showing a mixture of GFP- and RFP-positive cells. **(D,E)** Chimeric tumors on the mesentery have patches of green and red fluorescence. A clonal tumor that is only GFP-positive can be seen **(E)**, (arrow). **(F)** Endpoint of a mathematical simulation initialized with a mixed GFP/RFP spheroid of 56 cells attached to the mesothelial surface of the intestine. A 180 × 180 × 180 μm lattice (5.832 mm^3^) is partitioned into layers of smooth muscle (brown), extracellular matrix fibers (teal), mesothelium (dark blue), and vessel (red) creating a 0.84 mm^3^ tissue layer. Above the tissue is peritoneal fluid. The 3-D image shows a chimeric tumor (orange and green cells) after 7 days of growth.

It has been hypothesized that ovarian cancer cells aggregate and form spheroids when suspended in peritoneal fluid and that these spheroids then attach to the peritoneal surface and form large tumors (Shield et al., 2009). To test this hypothesis and to assess the clonality of the tumors at distinct sites, a mixture of SKOV3.ip1-GFP and SKOV3.ip1-RFP cells were co-injected into nude mice as a single cell suspension. The resulting tumors were imaged 2 weeks later. Tumors on the omentum developed as well-mixed chimeras, with both green and red fluorescence throughout (Figures 2A,B). In higher magnification images, small areas with predominantly red fluorescence can be distinguished from areas with predominantly green fluorescence, but there are no large sections of tumor expressing a single fluorescent protein (Figure 2C). The majority of the observed mesenteric tumors (92 ± 2% of 51 observed tumors) also have mixed green and red fluorescence, indicating that even these small tumors originated from a mixed spheroid (Figures 2D,E). The few small tumors consisting solely of GFP-positive or RFP-positive cells may represent rare instances where a single cell, or a small group of singly fluorescent cells, was able to adhere and grow (Figure 2E, arrow).

These data provided the first essential parameters for initialization of our OvTM mathematical model, since adhesion is a predominant feature of the cellular Potts framework (Graner and Glazier, 1992). Simulations were initiated with an adherent spheroid on the surface of the intestine. The spheroid subsequently pushes through the mesothelium. This process has been observed *in vitro*, where tumor spheroids cause retraction of the mesothelium using a myosin-mediated process (Iwanicki et al., 2011).

**Figure 3:**
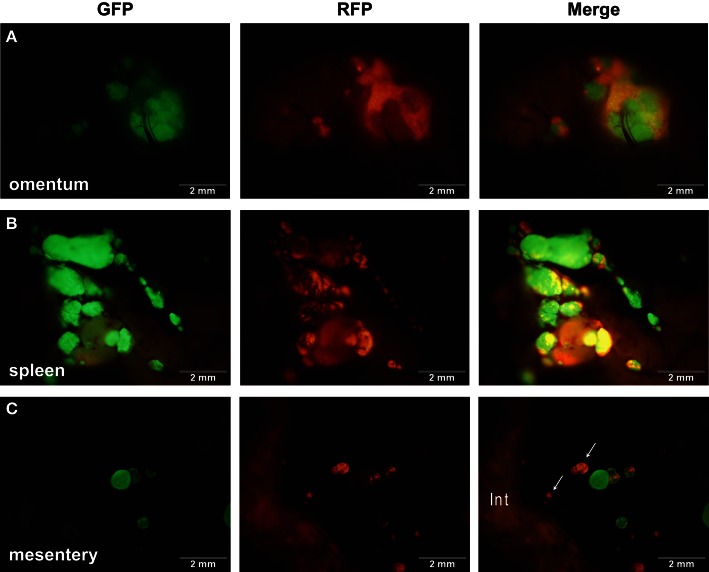
**SKOV3.ip1-GFP and RFP cells injected sequentially emphasize the importance of tumor cell-cell adhesion**. About 2.5 million SKOV3.ip1-GFP cells were injected into nude mice and allowed to grow for 1 week before injection of an equal number of SKOV3.ip1-RFP cells. Tumors were imaged 1 week later. **(A)** SKOV3.ip1-RFP cells preferentially adhere to and coat existing GFP-positive tumors on the omentum. **(B)** Similar to the omentum, SKOV3.ip1-RFP cells preferentially adhere to existing GFP-positive tumors on the spleen. **(C)** On the mesentery, RFP-positive cells form new tumors (white arrows) as well as adhering to existing tumors. Int, small intestine (autofluorescent).

The *in silico* experiments using OvTM showed that only by assigning high homotypic adhesion strength to the cancer cells could the model reproduce spheroid cohesiveness and growth (Figure 2F). For details on how the adhesion parameters were optimized, see Section “Materials and Methods” (Table 3). Simulations in which cancer cells adhered more strongly to other cancer cells than to any other cell type, produced the most rounded tumor morphology that closely resembled the xenograft tumors. Adding a cellular surface area constraint maintained the integrity of the cells themselves. Since the model indicated that tumor cell homotypic adhesion is an essential feature governing dissemination and growth, this concept was further tested experimentally by sequentially introducing fluorescent cancer cells into the peritoneum (Figure 3). In this experiment, tumors were first established by injection of 2.5 million SKOV3.ip1-GFP cells. After a period of 1 week to permit engraftment of the green-fluorescent cells, an equal number of SKOV3.ip1-RFP cells were injected. Following another week, the relative distribution and burden of both green and red fluorescent tumor cells were evaluated. As expected, SKOV3.ip1-GFP tumors formed on the omentum, mesentery, and spleen. Notably, the majority of red fluorescent cells adhered to and enveloped the pre-existing GFP-positive tumors as can be seen on the omental tumors (Figure 3A) and on the spleen (Figure 3B), rather than forming tumors independently. Although some exclusively RFP-positive tumors were present on the mesentery (Figure 3C, arrows), 70% of the 24 RFP-positive tumors examined were also GFP-positive.

### Tumor cells migrate through the open architecture of the mesentery in response to chemotactic signals

**Figure 4:**
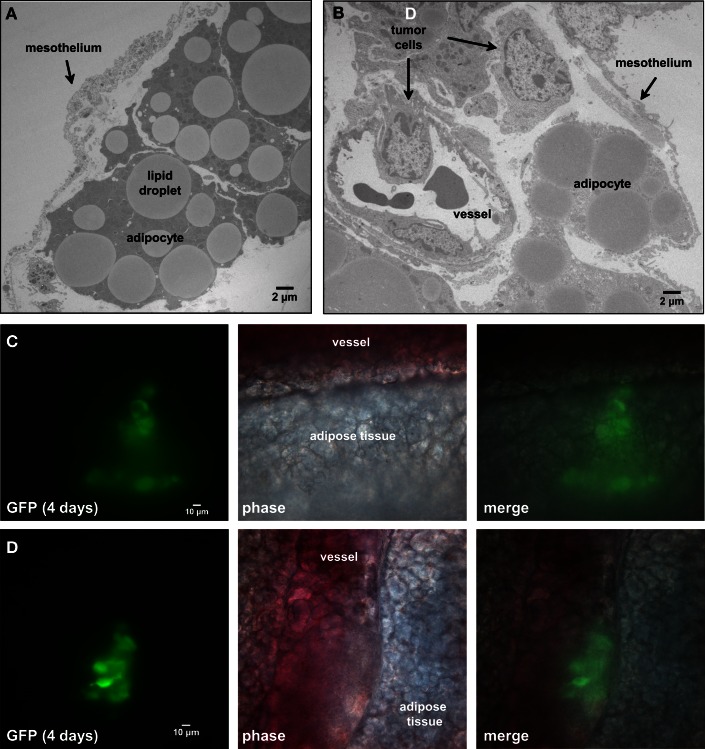
**Four days post-injection, tumor cells have invaded the mesentery and migrated through adipose tissue to approach mesenteric blood vessels**. **(A)** Transmission electron micrograph of the edge of the mesentery from a nude mouse. The mesentery architecture is open with loosely connected adipose cells below the mesothelium. Adipocytes are identified by their large lipid droplets. **(B)** Transmission electron micrograph of mesentery excised 4 days post-injection of SKOV3.ip1 cells. Arrows mark the locations of probable cancer cells. The cancer cells lie close to a blood vessel. **(C)** Cancer cells invading mesenteric adipose tissue adjacent to a vessel. On the right, GFP fluorescence of the cancer cells; middle, brightfield; left, composite image. **(D)** Cancer cells closely opposed to a mesenteric vessel. Panels are arranged as in **(C)**.

Our next goal was to identify specific features of the microenvironment that underlie differences in ovarian tumor morphology by examining the structure of external tissue layers facing the peritoneum. Both normal and tumor-associated tissues were extracted and prepared for TEM imaging. The micrograph in Figure 4A illustrates the open architecture of the normal mesentery, with loosely packed fat cells below the mesothelium. The mesothelial layer is remarkably thin in some areas, ranging from 0.4 to 2.5 μm in thickness. A second image (Figure 4B) shows a cross-section of mesentery excised from mice engrafted with SKOV3.ip1 human ovarian cancer cells. Labels mark the locations of probable tumor cells, identified by the characteristic ultrastructure of their nuclei. The tumor cells are interior to the mesothelium and adjacent to a small blood vessel.

To understand how tumors are established in the mesentery, SKOV3.ip1-GFP cells were injected into the peritoneum and 4 days later segments of the mesentery were removed for whole mount imaging. As shown in Figures 4C,D, images from this early time point provide a window into the initial steps in the engraftment process. Small groups of fluorescent cancer cells were seen within the mesenteric adipose layer (Figure 4C) or beneath the adipose layer immediately adjacent to vessels (Figure 4D).

We next used OvTM to evaluate the conditions necessary for cancer cells to migrate to mesenteric vessels within this short time period. The cellular Potts model is particularly well suited for this type of modeling, since it specifically represents cell–cell interactions and cell movement, which is governed by local contacts and chemotactic gradients. The 3-D stochastic model was populated with heterogeneous cell types (cancer cells, adipocytes, endothelial cells, and mesothelium) and geometric features (shape, location, organization, and thickness of tissue layers) based on TEM images. Extracellular factors, such as chemokines and oxygen, are described by diffusion equations with sources and sinks. In each case, cancer cells push through the mesothelial layer and degrade the underlying ECM, as shown experimentally (Sodek et al., 2008). Simulations can then demonstrate the extent of tumor invasion in response to different chemotactic environments.

**Figure 5:**
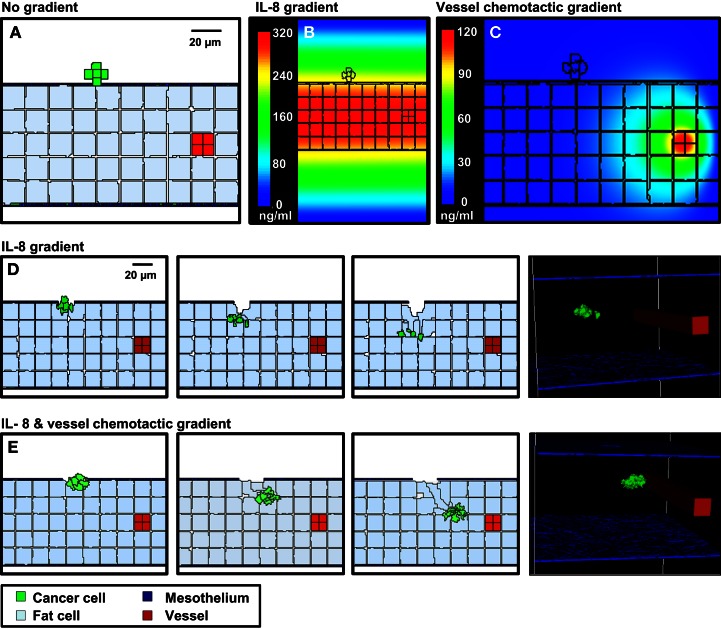
**3-D simulations of cancer cell attachment and migration to the mesenteric vessel**. **(A)** The initial configuration of the simulation has a seven-cell spheroid attached to the surface of the mesentery. To model this environment, a 170 × 170 × 263 μm lattice (7.6 mm^3^) is partitioned into five layers of adipocytes (light blue) sandwiched between single layers of mesothelium (dark blue) and ECM (teal) creating a 2.6 mm^3^ tissue layer surrounded by peritoneal fluid. A blood vessel on the right is represented by a solid rod (red). In the absence of chemotactic signals, the spheroid penetrates only the thin mesothelial layer at 1–2 min of simulation. **(B,C)** Steady-state distributions of chemotactic factors tested in these simulations. The color scale represents variation in factor concentrations in ng/ml. **(B)** The IL-8 gradient created by secretion of IL-8 from adipocytes. **(C)** A chemotactic gradient based on secretion of a hypothetical chemotactic factor (Chemotactic Factor 2) from mesenteric vessels. **(D)** Sequences in the simulation where a chemotactic gradient based on IL-8 is originating from the adipocytes. The spheroid migrates toward the center of the adipose layer. **(E)** Sequences of a simulation where chemotactic signals originate from both the adipocytes and the vessel. The spheroid migrates through the adipose layer toward the vessel.

Figure 5 shows results from three scenarios that were considered in these simulations. Movies for representative simulations are provided in Supplementary Data (Movies S1–S3 in Supplementary Material). For each case, a spheroid of seven cancer cells was initially positioned on a 3-D geometrical model of the mesentery. Mesothelial cells form the boundary with the peritoneum; adipocytes are dispersed within the interior, and a single vessel transverses the tissue.

In the first scenario, there is no local production of chemotactic factors imposed. The spheroid is positioned such that it is in contact with the ECM. In the absence of chemotactic factors, the spheroid dissolves the ECM and presses into the adipose tissue after 2 days (Figure 5A). This progression is too slow to explain cancer cell localization near mesenteric blood vessels in the mouse model.

SKOV3.ip1 cells have been shown to home toward chemokine-producing adipocytes and upregulate the IL-8 receptor (CXCR1) when co-cultured with adipocytes (Nieman et al., 2011). Therefore, in the second scenario, simulation parameters were modified such that all adipocytes within the mesentery secrete IL-8 at a rate of 2.2 × 10^−4^ pg/min/cell (Bruun et al., 2004), which diffuses at 1.5 × 10^4^ μm^2^/min (Li Jeon et al., 2002). Cancer cells are then allowed to chemotax up the resulting IL-8 gradient. In our model, spheroid invasion of the ultra-thin mesothelium is rapid, occurring at a rate of 10 μm/h based upon the *in vitro* experiments of Iwanicki et al. (2011). The pseudo-colored image in Figure 5B shows the predicted distribution of IL-8 in the mesenteric tissue at steady state, illustrating the initial conditions experienced by the tumor spheroid. Simulated IL-8 concentrations within the peritoneum agree with those measured experimentally (Barcz et al., 2002). When IL-8 chemotaxis is included in the simulation, the spheroid moves past the mesentery barrier and pushes between adipocytes to settle near the center of the adipose layer where the IL-8 concentration is greatest (Figure 5D). This occurs within 500 min after initialization. In this case, rapid chemotaxis occurs, but the cancer cells do not localize near the vessel.

In the final case, both adipose and endothelial cells are assumed to produce chemotactic factors that attract cancer cells. We introduce a new chemotactic factor (Chemotactic Factor 2) that originates from the mesenteric vessel. Steady-state values represented in the pseudo-colored profile in Figure 5C show that a significant gradient can be established by endothelial cell secretion of Chemotactic Factor 2 at a rate of 1.8 × 10^−4^ pg/min/cell, which is comparable to that of IL-8 secretion from the adipocytes, and assuming diffusion and decay rates similar to VEGF. When cells are arranged in this geometry, the presence of both chemotactic gradients causes spheroids to penetrate the mesothelial layer, move by chemotaxis through the loose adipose layer toward the vessel, and halt at the tightly adherent barrier of the vessel wall (Figure 5E). Together with the experimental data, these results support the conclusion that both adipocytes and endothelial cells are likely sources of chemokines that attract ovarian tumor cells.

### Small SKOV3.ip1 tumors attached to surfaces of the stomach or small intestine are non-invasive and initiate angiogenesis

**Figure 6:**
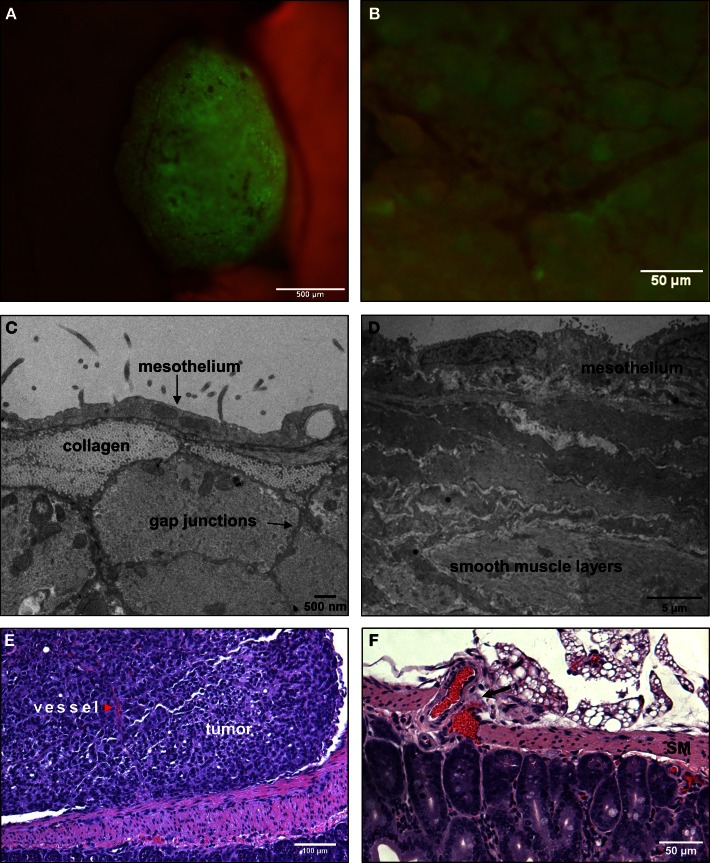
**Tumors that adhere to the walls of the small intestine are spherical and non-invasive**. **(A)** An SKOV3.ip1-GFP tumor adhering to the wall of the small intestine 2 weeks post-injection in an RFP nude mouse. Shown is a composite GFP/RFP image. Small vessels are visible on the surface of the tumor. **(B)** Higher magnification image of the vascular tree infiltrating a green-fluorescent tumor on the intestine. **(C)** Transmission electron micrograph of the small intestine wall. Tissue was collected from a nude mouse 4 days post-injection with SKOV3.ip1-GFP cells. The wall of the small intestine consists of a thin layer of mesothelium overlaying bundles of smooth muscle fibers. **(D)** TEM image of stomach ultrastructure, illustrating the distinct cellular layers. **(E)** An H&E-stained section of a tumor attached to the small intestine. There is a clear delineation between the intestine and the tumor. The tumor is vascularized (red arrowhead). **(F)**. An H&E-stained section of the surface of a mouse intestine. Arrow points to vessels at the intestine-mesentery junction.

We next focused on explanations for the distinct morphology of tumors attached to the stomach or small intestine, which do not invade the tissue and instead grow outward into the peritoneal cavity (Figure 6A). We again sought insight from high resolution TEM images. As shown Figure 6C (mesentery) and Figure 6D (stomach), the outer mesothelial layer remains relatively thin over these organs (typically 0.5 μ thick). The next layer is distinguished by dense collagen deposits. Prominent smooth muscle layers can be seen in both the small intestine and stomach, where the muscle cells are closely opposed and connected by gap junctions (Friend and Gilula, 1972) (arrows, Figure 6B).

The morphology of a tumor attached to the outer rim of the lower intestine is seen at a lower magnification in Figure 6E, which shows a representative section from a formalin-fixed, paraffin block stained with H&E (hematoxylin and eosin). The smooth muscle of the small intestine remains intact at the tumor/tissue interface. Thus, tumors attached to the exterior of the gut are presented with a discrete barrier and adapt by growth into the available and flexible space between organs. The intestine has a capillary bed that provides oxygen to the mesothelium and contributes to the oxygenated peritoneal environment (Figure 6F). Because of the lack of a smooth muscle barrier, these vessels may provide a more accessible endothelial source for neoangiogenesis critical to tumor success.

Microscopic evaluations provide support for this hypothesis (Figures 6B,E). Fluorescence imaging shows the remarkable extent of tumor vascularization, even in young GFP-positive tumors attached to the intestinal wall at 2 weeks post-engraftment (Figure 6B). A red arrowhead in Figure 6E points to a vessel visible within the tumor cross-section. This evidence led us to conduct simulations to explain the rapid onset of neovascularization in the absence of invasion.

### SKOV3.ip1 tumor spheroids are initially well oxygenated and likely induce neovascularization via constitutive secretion of angiogenic factors

**Figure 7:**
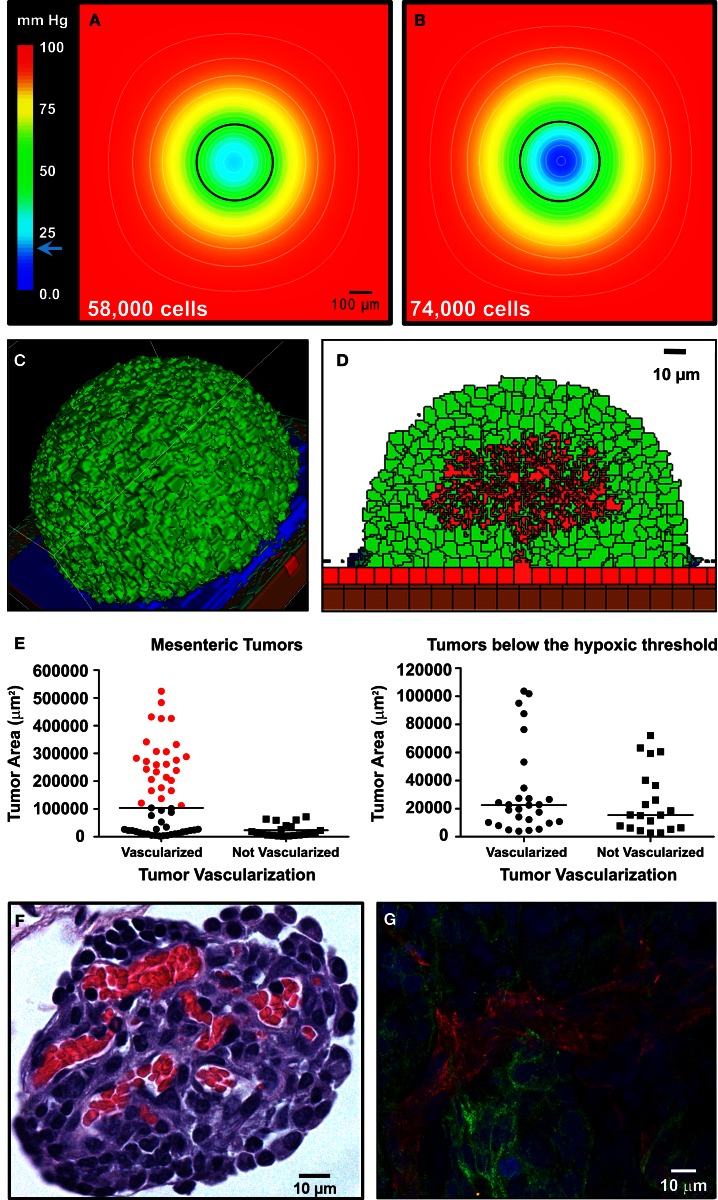
**Vascularization of tumors is rapid and can be attributed to constitutive release of angiogenic factors by SKOV3.ip1 cells**. **(A,B)** Representations of the steady-state oxygen gradients from coarse-grained simulations of spheroids suspended in peritoneal fluid. The color scale indicates the range of oxygen concentrations in mm Hg. Black circles mark the perimeter of the spheroids. **(A)** The oxygen gradient through the middle of a spheroid 336 μm in diameter (58,000 cells). At this size, all cells are well oxygenated with an oxygen partial pressure above the hypoxic threshold of 19 mm Hg (indicated on the color scale as a blue arrow). The lowest oxygen concentration at the core of the spheroid is 21.6 mm Hg. **(B)** The oxygen gradient through the middle of a spheroid 364 μm in diameter (74,000 cells). By the time a spheroid has reached this size, the core is hypoxic (0.5 mm Hg). **(C,D)** OvTM simulations of angiogenesis in a tumor attached to the intestinal wall, assuming constitutive release of VEGF from cancer cells. **(C)** 3-D image of the simulation after 7.8 days when the tumor has grown to 6,400 cells. **(D)** 2-D slice through the middle of the tumor in **(D)** to show vessel tree morphology. **(E)** Scatter dot plots of mesenteric tumor vascularization with respect to cross-sectional tumor area as determined from H&E-stained sections of mouse intestine and mesentery collected 3 weeks post-injection. Areas of all mesenteric tumors measured are plotted on the left. Tumors with areas above the predicted hypoxic threshold are in red. All non-vascularized tumors fall below the hypoxic threshold. The right plot shows the area and vascularization status of small tumors that fall below the hypoxic threshold. A majority of these small tumors (57%) are also vascularized. Lines indicate the median value. **(F)** Cross-sectional view of a small mesenteric tumor after H&E staining. Red blood cells (red) can be seen populating vessels within the tumor (blue). **(G)** Confocal image of ovarian tumor removed from the surface of the gut and labeled with anti-CD31 antibody (endothelial cell marker, red fluorescence) to distinguish tumor vasculature. An anti-GFP antibody with an FITC-labeled secondary antibody marks GFP-expressing cancer cells; Hoechst (blue fluorescence) labels the nuclei of all cells in the field of view.

For the *in silico* model of angiogenesis, micron-scale geometric parameters for the tissue surface architecture were again determined from TEM images. For the gut, adhesion between smooth muscle cells is set sufficiently high as to prevent spheroid penetration below the mesothelial and collagen layers. Under these constraints, simulations of tumor adhesion and growth result in the formation of spherical tumors that are consistent with the morphology of engrafted tumors on mouse intestine (compare Figures 6A and 7C). Since these simulations incorporate published values for oxygen content in the peritoneal fluid and oxygen diffusion rate (MacDougall and McCabe, 1967; Kizaka-Kondoh et al., 2009), it is possible to calculate the distribution of oxygen in all locations during tumor growth. By coarse-graining the model (1 voxel = 1 cell), we were able to determine the oxygen concentration gradients for large spheroids suspended in the peritoneal fluid. In spheroids of varying sizes, oxygen concentration decreased within the core. However, spheroids up to 336 μm in diameter (58,000 cells) approach the hypoxic threshold of 19 mm Hg of O_2_ (Höckel and Vaupel, 2001), but are not yet hypoxic at their core (Figure 7A). Continued growth to 364 μm in diameter (74,000 cells) results in a hypoxic core with an oxygen concentration of 0.5 mm Hg (Figure 7B), leading to the prediction that the hypoxic threshold is reached when the spheroid is between these two sizes. Tumor sizes in mouse samples were compared based on the cross-sectional area of tumors in H&E-stained sections. Mesenteric tumor vascularization with respect to tumor area is shown as scatter dot plots in Figure 7E. Of the 76 tumors measured, all tumors above the predicted hypoxic threshold (red) were vascularized. However, 57% of small tumors with an area of <104,000 μm^2^ (below the predicted hypoxic threshold) were also vascularized. These results suggest that angiogenesis is not solely hypoxia-driven in the SKOV3.ip1 model.

In the next series of simulations, we examined how angiogenesis might originate from these spheroids in the absence of hypoxic signaling. There is experimental evidence that SKOV3.ip1 cells constitutively express VEGF *in vivo* and *in vitro* even when maintained in well-oxygenated tissue culture conditions (Yoneda et al., 1998). Secretion of VEGF by the cancer cells was therefore incorporated into these simulations. Small spheroids attached to the gut penetrate the mesothelial layer, permitting VEGF secreted from cancer cells to initiate chemotactic gradients and attract endothelial cells that line blood vessels in the sub-mesothelial layer. The process of angiogenesis is driven by endothelial cell chemotaxis toward VEGF, and adhesive interactions between endothelial sprout cells and cancer cells. To produce vasculature visually similar to that in very small xenograft tumors, latent endothelial cells must begin to proliferate and migrate as soon as the spheroid comes close enough to the vessel to allow diffusion of low concentrations of VEGF. Given spheroid VEGF production of 3.82 × 10^−7^ pg/min/SKOV3.ip1 cell, the threshold for the switch from latent to sprouting endothelial cells was set at 2.08 × 10^−8^, to initiate angiogenesis when the spheroid is ∼5 μm from the vessel.

Angiogenesis in the OvTM model follows validated methods that treat VEGF as diffusible molecules whose gradient, together with the biophysical environment, drives endothelial migration and proliferation, and eventually morphogenesis of vessel sprouts (Bauer et al., 2007; Shirinifard et al., 2009). Parameters and model assumptions are described in the Materials and Methods. Simulation results show that constitutive production of VEGF from even a small spheroid of SKOV3.ip1 cells should be capable of initiating vascular outgrowths that penetrate the spheroid within 12 h of attachment (Figure 7D; Movie S4 in Supplementary Material). Results of the computational model are consistent with our experimental observations that even very small tumors, comprised of less than 20,000 SKOV3.ip1 cells, are fully vascularized (Figure 7F). They are also consistent with 3-D images of tumor slices stained for confocal fluorescence imaging that show extensive tumor vascularization 3 weeks post-injection (Figure 7G). In this final image, an anti-CD31 antibody (red) marks endothelial cells, Hoechst (blue) stains the cell nuclei and an anti-GFP antibody (green) labels the GFP-positive cancer cells. A rotating 3-D view of this tumor section is found in Movie S5 in Supplementary Material.

## Discussion

In this work, we combine a murine xenograft model with a computational model, OvTM, to evaluate critical factors governing the dissemination and growth patterns of ovarian cancer in the peritoneum. These models best represent ovarian cancer relapse after debulking surgery, where disease progression initiates from microscopic residual disease in the peritoneal chamber.

In cellular Potts models, the variety of constraints placed on cells must be properly balanced to produce biologically reasonable cellular and tissue structure and movement. We quantitatively modeled oxygenation in a simple spheroid to estimate at what diameter the tumor center would become hypoxic, for comparison with diameters of vascularized tumors observed in the mouse xenografts. Cancer cell homotypic and heterotypic adhesions on smooth muscle were then tuned to regenerate the spheroidal morphology of SKOV3.ip1 xenograft tumors on the small intestine or stomach (Figure 6A). To simulate the depth to which spheroids penetrate in soft tissue due to a chemotactic motive force, the underlying tissue structure was adjusted to represent a fatty section of the mesentery. In the simulations, tumors remain on the surface of smooth muscle, a tissue with many underlying tight junctions (Figures 6C,D), and invade soft tissues with space between cells, such as the mesentery (Figure 4A).

Finally, we simulated VEGF-driven angiogenic morphogenesis borrowing from previous methods using cellular Potts models for tumor-driven angiogenesis (Bauer et al., 2007, 2009; Shirinifard et al., 2009), in which endothelial cell chemotaxis toward soluble VEGF leads to angiogenic sprouting and branching. These models treated VEGF as a diffusible molecule whose gradient, together with the biophysical environment, drives endothelial migration and proliferation, and eventually morphogenesis of vessel sprouts. We did not include other angiogenesis dynamics from Bauer et al. (2009), which considered endothelial cell interactions with the ECM to further drive sprouting morphogenesis, nor does our model assess tumor growth as in Shirinifard et al. (2009), which modeled tumor growth in response to a growing network of surrounding vessels providing nutrients. Instead, angiogenesis was modeled as a simple morphogenetic process driven by chemotaxis and differential adhesion that penetrates a 3-D tumor. The sprouting vessel’s base cell (or cells) has a semi-permanent elastic bond to the existing vessel, describing the labile adhesion interactions between them. Otherwise, differential adhesions between tumor and endothelial cells facilitate endothelial sprouting up the VEGF gradient.

The basic OvTM model and parameter set remain the same for all three groups of simulations (spheroids on muscle, spheroids invading mesenteric fat, and angiogenic sprouts in spheroids on muscle), except for the following: Between the cases of spheroid growth in different niches, the tissue surface is comprised of different cell types with their associated parameters. In angiogenesis simulations, the volume constraint for cancer cells was increased to help prevent cell fragmentation during migration. Constraints on endothelial cells in the angiogenesis model (proliferating endothelial, non-proliferating endothelial, and permanent vessel) include elastic labile adhesion bonds between each type of vascular cell and its neighbors. The CC3D simulation code will be available upon request to the authors.

We show that, for the aggressive SKOV3.ip1 cell line, homotypic adhesion between cancer cells is a defining feature that favors the aggregation of cancer cells into small spheroids. The spheroid morphology may promote adhesion-mediated cell survival signals and allow cancer cells to evade anoikis, a cell death program usually triggered by loss of cell adhesion to the ECM (Kim et al., 2012). We speculate that these strong homotypic interactions may help to explain the typical clinical presentation where ovarian cancer is largely confined to the peritoneum and often accompanied by ascites. It is notable that others have shown spheroids are less susceptible to chemotherapeutic agents and may therefore contribute to relapse (Shield et al., 2009).

While some treatment regimens have focused on reducing metastatic spread through limiting cancer cell adhesion to the mesothelium (Sawada et al., 2012), we were interested in whether interactions between cancer cells might also be an important target. Using serial injections of green-fluorescent and red-fluorescent cells, we demonstrated that newly introduced cancer cells preferentially adhere to existing tumors. Strong cell-cell adhesion between cancer cells that stabilizes tumor clusters in the simulations could explain this observation. However, autocrine factors may also contribute to cancer cell homing, similar to the release of IL-6 and IL-8 from breast tumors that draws circulating tumor cells back to the primary tumor site (Kim et al., 2009). Although the mechanism is not well understood, therapies targeting ovarian cancer cell-cell homotypic adhesion may be worth consideration. In addition to limiting tumor mass, such drugs might be administered in combination with conventional chemotherapy to improve drug penetration.

Distinct niches within the peritoneal microenvironment also help to restrict cancer cells to the peritoneum and limit metastatic spread to other anatomical sites. Based upon the animal and mathematical models, colonization and growth is favored in loosely organized tissues. There are similarities between the open architectures of the mesentery and omentum, two organs that are colonized by SKOV3.ip1 cells and share rich beds of adipose tissue known to secrete cytokines and growth factors attractive to cancer cells (Collins et al., 2009; Klopp et al., 2012). The adipocyte-rich omentum has a slightly thicker mesothelial layer than the mesentery, but has stomata or openings in the mesothelium above the milky spots that expose the underlying layers (Cui et al., 2002). The open architecture of these organs offers few barriers to cancer cells that undergo chemotaxis in response to local chemokine production. In contrast, even the aggressive SKOV3.ip1 cancer cell line is largely blocked by physical barriers such as the smooth muscle layers in the GI tract.

Our work is consistent with recent studies by Nieman et al. (2011), who showed that SKOV3.ip1 cells adhere to the omentum as early as 20 min post-injection and migrate in response to IL-8 and other chemotactic agents produced by adipocytes. In addition, we provide new evidence suggesting that SKVO3.ip1 cells migrate through the mesothelium and adipose tissue toward mesenteric vessels. Chemotaxis of cancer cells toward existing vessels has been observed in rodent models injected subcutaneously with mammary carcinoma cells (Li et al., 2000). Based on results from OvTM simulations, we propose that a chemotactic factor originating from the vessel may mediate this process. Although the identity of the factor is unknown, it is possible that vessels also produce an IL-8 gradient that attracts cancer cells, since activated vascular smooth muscle cells are capable of producing IL-8 (Wang et al., 1991). Growth factors secreted by perivascular tumor-associated macrophages, such as the epidermal growth factor (EGF), could also promote local survival and proliferation of tumors that take up residence near vessels (Lewis and Pollard, 2006).

Interestingly, rodent models that showed tumor migration to blood vessels also exhibited early angiogenesis in tumors consisting of fewer than 300 cells (Li et al., 2000). The constitutive expression of angiogenic factors by SKOV3.ip1 cells may be the single most important feature contributing to the aggressive growth of this cancer cell line after engraftment. In preliminary data not shown, microarray studies showed that VEGF mRNA levels differ less than twofold in cultured SKOV3.ip1 cells versus *in vivo*. In OvTM simulations, this modest level of constitutive production is sufficient for even minute tumor spheroids to recruit endothelial cells from nearby vessels (Figure 7). This result is in contrast to the classical solid tumor situation, where angiogenesis is initiated only after the interior tumor cells become hypoxic and upregulate VEGF production (Shweiki et al., 1992; Pugh and Ratcliffe, 2003). In patients, however, it is important to note that the balance of constitutive and induced production of angiogenic factors by cancer cells may vary widely. Therefore, assessment of angiogenic factor transcriptional profiles or direct measurement of angiogenic factor levels in serum/cystic fluid may be critical to identify patients at risk for relapse, a concept that has also been proposed by others (Harlozinska et al., 2004; Li et al., 2004).

## Conflict of Interest Statement

The authors declare that the research was conducted in the absence of any commercial or financial relationships that could be construed as a potential conflict of interest.

## Supplementary Material

The Supplementary Material for this article can be found online at http://www.frontiersin.org/Molecular_and_Cellular_Oncology/10.3389/fonc.2013.00097/abstract
